# An Ensemble Learning Framework for Detecting Protein Complexes From PPI Networks

**DOI:** 10.3389/fgene.2022.839949

**Published:** 2022-02-24

**Authors:** Rongquan Wang, Huimin Ma, Caixia Wang

**Affiliations:** ^1^ School of Computer and Communication Engineering, University of Science and Technology Beijing, Beijing, China; ^2^ School of International Economics, China Foreign Affairs University, Beijing, China

**Keywords:** protein complexes, protein-protein interaction networks, graph clustering algorithms, ensemble learning, network embedding, biological information

## Abstract

Detecting protein complexes is one of the keys to understanding cellular organization and processes principles. With high-throughput experiments and computing science development, it has become possible to detect protein complexes by computational methods. However, most computational methods are based on either unsupervised learning or supervised learning. Unsupervised learning-based methods do not need training datasets, but they can only detect one or several topological protein complexes. Supervised learning-based methods can detect protein complexes with different topological structures. However, they are usually based on a type of training model, and the generalization of a single model is poor. Therefore, we propose an Ensemble Learning Framework for Detecting Protein Complexes (ELF-DPC) within protein-protein interaction (PPI) networks to address these challenges. The ELF-DPC first constructs the weighted PPI network by combining topological and biological information. Second, it mines protein complex cores using the protein complex core mining strategy we designed. Third, it obtains an ensemble learning model by integrating structural modularity and a trained voting regressor model. Finally, it extends the protein complex cores and forms protein complexes by a graph heuristic search strategy. The experimental results demonstrate that ELF-DPC performs better than the twelve state-of-the-art approaches. Moreover, functional enrichment analysis illustrated that ELF-DPC could detect biologically meaningful protein complexes. The code/dataset is available for free download from https://github.com/RongquanWang/ELF-DPC.

## 1 Introduction

Most complex systems, such as biological systems and human society, can be presented as complex networks in the real world. Social networks, biological networks, brain networks, citation networks, and protein-protein interaction networks are examples of complex networks (Pourkazemi and Keyvanpour, 2017). Community detection in complex networks is essential in many fields, aiming to identify clusters with high internal connectivity. These clusters are well separated from the rest of the network. Over the past several years, the study of community identification in complex networks has grown popular. Community detection is a fundamental problem in network analysis that tries to mine the hidden structure of a specific complex network (Fortunato, 2010; Abduljabbar et al., 2020). In bioinformatics, the crucial topic is to mine protein complexes in PPI networks. Proteins usually interact with each other, forming protein complexes to accomplish their biological functions (Gavin et al., 2002; Spirin and Mirny, 2003). As a community structure in the PPI network, it may be the natural protein complex, and the proteins in the protein complex should be highly interconnected (Girvan and Newman, 2002; Chen et al., 2014). The truth is that the prediction of protein complexes is essential for studying cellular organization theory and understanding protein complex formation. Biologically, a protein complex is a group of proteins formed by interacting simultaneously and in place. The detection of protein complexes using biological experiments is both costly and time-consuming. With the development of high-throughput experimental methods, many PPI networks have been produced, which usually have small world, scale-free, and modularity characteristics. They could be formulated as graphs where the nodes represent the proteins, and the edges represent the interactions. Therefore, many computational algorithms present alternate ways to automatically discover protein complexes from the PPI networks. More details on the related work are introduced in the related work section.

### 1.1 Related Work

During the past decade, various computational methods have been presented to identify protein complexes in PPI networks. We will briefly review the related work from three aspects. The first is identifying protein complexes based on unsupervised learning-based methods. Another type of identifying protein complex methods is based on a model optimization-based method. The last type of identifying protein complex methods is based on supervised learning-based methods.

#### 1.1.1 Unsupervised Learning-Based Methods

Many researchers hypothesize that subgraphs with different topological structures in PPI networks are factual protein complexes (Wang et al., 2010) such as density, k-clique, and core-attachment structures. Most of these methods are either global heuristic search, local heuristic search, or both. Meanwhile, some methods integrate topological and biological information to further improve the accuracy of detecting protein complexes.

Many local heuristic-based methods have been proposed to identify protein complexes. For instance, Altaf-Ul-Amin et al. (Altaf-Ul-Amin et al., 2006) developed DPClus, which generates clusters by ensuring density and checking the periphery of the clusters. Gavin et al. (Gavin et al., 2006) studied the organization of protein complexes, demonstrating that a protein complex generally contains a unique protein complex core and attachment proteins, called a core-attachment structure. Here, proteins in a protein complex core have relatively more reliable interactions among themselves. The attachment proteins are the surrounding proteins of the protein complex core to assist it in performing related functions (Lakizadeh et al., 2015). Wu et al. (Wu et al., 2009) proposed a classic protein complex discovery method (COACH) using the core-attachment structure. COACH first detects protein complex cores and then identifies its attachment proteins to form a whole protein complex. Peng et al. (Peng et al., 2014) designed a PageRank Nibble strategy to give adjacent proteins different probabilities with core-attachment structures and proposed WPNCA to predict protein complexes. Nepuse et al. (Nepusz et al., 2012) presented ClusterONE, which utilizes a demanding growth process to mine subgraphs with high cohesiveness that may be protein complexes. Recently, Wang et al. (Wang et al., 2020) presented a new graph clustering method using a local heuristic search strategy to detect static and dynamic protein complexes. These local heuristic methods have strong local searchability, but finding an optimal global solution is difficult.

Meanwhile, some global heuristic-based methods have been proposed to identify protein complexes. In 2009, Liu et al. (Liu et al., 2009) used an iterative method to weight PPI networks and developed a maximal clique-based method (CMC) to discover protein complexes from weighted PPI networks. Wang et al. (Wang et al., 2012) were inspired by the hierarchical organization of GO annotations and known protein complexes. Then they proposed OH-PIN, which is based on the concepts of overlapping M-clusters, *λ*-module, and clustering coefficients to detect both overlapping and hierarchical protein complexes in PPI networks. PC2P (Omranian et al., 2021) is a parameter-free greedy approximation algorithm casts the problem of protein complex detection as a network partitioning into biclique spanned subgraphs, which include both sparse and dense subgraphs. Although these global heuristic search methods have a strong global search ability, they require considerable time and computing resources.

Recently, some methods based on network embedding strategies have been used to detect protein complexes. DPC-HCNE (Meng et al., 2019) is a novel protein complex detection method based on hierarchical compressing network embedding and core-attachment structures. It can preserve both the local topological information and global topological information of a PPI network. CPredictor 5.0 (Yao et al., 2019) uses the network embedding method Node2Vec (Grover and Leskovec, 2016) to learn node feature vector representation and then calculates the node embedding similarity and the functional similarity between interacting proteins to construct the weight PPI networks. These methods illustrate that employing the network embedding method could improve the accuracy of protein complex identification.

It is well known that PPI networks contain many false-positive and false-negative interactions, i.e., noise. To overcome the noise of the PPI networks, some studies try to exploit biological information, such as gene expression data (Keretsu and Sarmah, 2016), gene ontology (GO) data (Wang et al., 2019; Yao et al., 2019), and subcellular localization data (Lei et al., 2018) to complement the interactions in PPI networks. CPredictor2.0 (Xu et al., 2017) effectively detects protein complexes from PPI networks, and first groups proteins based on functional annotations. Then, it applies the MCL algorithm to detect dense clusters as protein complexes. Zhang et al. (Zhang et al., 2016) calculated the active time point and the active probability of each protein and constructed dynamic PPI networks. Then a novel method was proposed based on the core-attachment structure. Zhang et al. (Zhang et al., 2019) proposed a novel method based on the core-attachment structure and seed expansion strategy to identify protein complexes using the topological structure and biological data in static PPI networks. ICJointLE (Zhang et al., 2019) is a novel method to identify protein complexes with the features of joint colocalization and joint coexpression in static PPI networks. NNP (Zhang et al., 2021) is a new method for recognizing protein complexes by topological characteristics and biological characteristics. Some methods (Zaki et al., 2013; Wang et al., 2019) are based on topological information to weight interactions in PPI networks. For example, PEWCC (Zaki et al., 2013) is a novel graph mining method that first assesses the reliability of the interactions and then detects protein complexes based on the concept of the weighted clustering coefficient. These methods have shown that the accuracy of protein complex identification can be significantly improved by integrating network topological structure and multiple biological information.

#### 1.1.2 Model Optimization-Based Methods

Several recent methods suggested that identifying protein complexes or community structures can be an optimization problem using network topology and protein attributes. For example, RNSC (King et al., 2004) attempts to find an optimal set of partitions of a PPI network graph by employing different cost functions for detecting protein complexes. RSGNM (Zhang et al., 2012) is a regularized sparse generative network model that adds another process that generates propensities into an existing generative network model for protein complex identification. EGCPI (He and Chan, 2016) formulates the problem as an optimization problem to mine the optimal clusters with densely connected vertices in the PPI networks to discover protein complexes. DPCA (Hu et al., 2018) formulates the problem of detecting protein complexes as a constrained optimization problem according to protein complexes’ topological and biological properties. In particular, it is an algorithm with high efficiency and effectiveness. GMFTP (Zhang et al., 2014) is a generative model to simulate the generative processes of topological and biological information, and clusters that maximize the likelihood of generating the given PIN are considered protein complexes. DCAFP (Hu and Chan, 2015) transforms the problem of identifying protein complexes into a constrained optimization problem and introduces an optimization model by considering the integration of functional preferences and dense structures. He et al. (He et al., 2019) introduced a novel graph clustering model called contextual correlation preserving multiview featured graph clustering (CCPMVFGC) for discovering communities in graphs with multiview features, viewwise correlations of pairwise features and the graph topology. VVAMo (He et al., 2021a) is a novel matrix factorization-based model for communities in complex network. It proposes a unified likelihood function for VVAMo and derives an alternating algorithm for learning the optimal parameters of the proposed model. In 2017, Zhang et al. (Zhang et al., 2017) proposed a new firefly clustering algorithm for transforming the protein complex detection problem into an optimization problem. IMA (Wang et al., 2021) is a novel improved memetic algorithm that optimizes a fitness function to detect protein complexes. These model optimization-based methods usually have more parameters and variables, and the parameter optimization process is time-consuming. However, these methods also have some significance for us to transform the identification of protein complexes into an optimization problem.

#### 1.1.3 Supervised Learning-Based Methods

The methods mentioned above are either unsupervised learning-based or model optimization-based methods that identify protein complexes using predefined assumptions and determined models. Unsupervised learning-based methods do not need to resolve practical problems, such as insufficient feature extraction from known protein complexes, model selection, and model training. Those methods cannot utilize the information of known protein complexes, and they neglect some other topological protein complexes such as the ‘star’ mode and ‘spoke’ mode and so on. Generally, supervised learning-based methods first train a supervised learning model by extracting features, and then trained supervised learning models are used to search new protein complexes.

Many standard protein complex datasets have been obtained in recent years. Therefore, several supervised learning-based methods based on training regression or classification models are proposed to discover protein complexes from PPI networks. For example, Qi et al. (Qi et al., 2008) proposed a framework to learn the parameters of the Bayesian network model for discovering protein complexes. Yu et al. (Yu et al., 2014) presented a supervised learning-based method to detect protein complexes, which used cliques as initial clusters and selected a trained linear regression model to form protein complexes. Lei et al. (Shi et al., 2011) proposed a semisupervised algorithm, and trained a neural network model to detect protein complexes. ClusterEPs (Liu et al., 2016) estimated the possibility of a subgraph being a protein complex by emerging patterns (EPs). Dong et al.(Dong et al., 2018) provided the ClusterSS method, which integrates a trained neural network model and local cohesiveness function to guide the search strategy to identify protein complexes. Liu et al. (Liu et al., 2018) proposed a supervised learning method based on network embeddings and a random forest model for discovering protein complexes. Based on the decision tree, Sikandar et al. (Sikandar et al., 2018) presented a method using biological and topological information to detect protein complexes. Liu et al.(Liu et al., 2021) proposed a novel semisupervised model and a protein complex detection algorithm to identify significant protein complexes with clear module structures from PPI networks. Mei et al. (Mei, 2022) proposed a computational method that combines supervised learning and dense subgraph discovery to predict protein complexes. On the one hand, the accuracy of these detection methods based on semisupervised learning or supervised learning is limited due to the small training dataset. On the other hand, these methods only train a single type of learning model, so these models are not so generalizable and their learning ability has certain limitations.

Some existing studies show that graph neural networks (GNNs) methods can effectively learn graph structure and node features. For example, Kipf et al. (Kipf and Welling, 2016) presented a scalable approach for semisupervised learning on graph-structured data. The proposed graph convolutional network (GCN) model is based on an efficient variant of convolutional neural networks. It can encode both graph structure and node features in a way useful for semisupervised classification. In 2021, Zaki et al. (Zaki et al., 2021) introduced various GCN approaches to improve the detection of protein complexes. graph attention networks (GATs), which aggregate neighbor nodes through the attention mechanism, realize the adaptive allocation of weights of different neighbors, thus greatly improving the expression ability of GNN models. He et al. (He et al., 2021b) proposed a class of novel learning-to-attend strategies, named conjoint attentions (CAs) to construct graph conjoint attention networks (CATs) for GNNs. CAs offer flexible incorporation of layerwise node features and structural interventions that can be learned outside the GNNs to compute appropriate weights for feature aggregation. We will study the detection of protein complexes in PPI networks using GATs in the future.

### 1.2 Observations and Contributions

Based on the related work, assigning weights to the interacting edges by the network embedding method and multiple biological information can effectively improve the accuracy of the detection methods. Meanwhile, some studies have shown that protein complexes have core-attachment structures. Therefore, our ELF-DPC is based on a core-attachment structure, and we constructed a weighted PPI network. Second, we proposed a protein complex core strategy to mine local protein complex cores. We identified global protein complex cores using the CPredictor2.0 method, which endows our ELF-DPC with both global search ability and local search ability. Third, most current methods are based on either unsupervised learning or supervised learning. Unsupervised learning-based methods can detect only one or several topological protein complexes and cannot fully learn the characteristics of known protein complexes. Supervised learning-based methods can learn the characteristics of known protein complexes, detecting protein complexes with different topological structures. Still, current supervised learning-based methods are based on a single base model for training. However, the generalization of a single model is poor. Therefore, we propose an ensemble learning model consisting of a trained voting regression model based on different types of base regression models and structural modularity to detect protein complexes with different topological structures. Finally, we proposed a graph heuristic search strategy to extend each protein complex core to form a protein complex. The results obtained show that ELF-DPC attained superior performances over 12 state-of-the-art methods. Furthermore, functional enrichment analysis results of ELF-DPC showed higher biological relevance by GO enrichment analysis.

To summarize, we make the following contributions:

• We introduce a protein complex core mining strategy based on the core-attachment structure and design a graph heuristic search strategy to search protein complexes.
• We propose structural modularity to describe the inherent topological organization of protein complexes.
• We present some new topological features and design an ensemble learning model by combining structural modularity and a voting regression model, which quantifies the possibility for a cluster as a protein complex.
• We present an ensemble learning framework to identify protein complexes, and it achieves better performance than other competing methods.

The rest of this study is organized as follows. The Materials and methods section introduces the datasets, terminologies, and methods. The Experiments and results section describes evaluation metrics and parameter selection and compare ELF-DPC with the competing methods. Finally, the Conclusion section provides a conclusion and future work.

## 2 Materials and Methods

### 2.1 Datasets

#### 2.1.1 Protein-Protein Interaction Networks

In this paper, we used the four PPI networks for the experiments, i.e., Gavin (Gavin et al., 2006), Krogan core (Krogan et al., 2006), DIP (Xenarios et al., 2002), and MIPS (Güldener et al., 2006). The detailed properties of these PPI networks are shown in Table 1. Here, the self-interactions and duplicate interactions were eliminated.

**TABLE 1:** The detailed properties of the protein-protein interaction datasets.

Dataset	Number of node	Number of edge	Density
Gavin	1855	7,669	0.004 459 796 985
Krogan core	2,674	7,075	0.001 979 684 934
DIP	4,930	17 201	0.001 415 721 912 41
MIPS	4,553	12 318	0.001 188 694 605 27

#### 2.1.2 Standard Protein Complexes

We used two standard protein complexes that were constructed in the literature (Wang et al., 2020). Their properties are shown in Table 2. Here, standard protein complexes 1 consists of the known protein complexes from MIPS (Mewes et al., 2004), SGD (Hong et al., 2007), TAP06 (Gavin et al., 2006), ALOY (Aloy et al., 2004), CYC 2008 (Pu et al., 2009), and NEWMIPS (Friedel et al., 2009). Standard protein complexes 2 is also a combined protein complex dataset (Ma et al., 2017). It consists of the Wodak database (Pu et al., 2009), PINdb and GO complexes (Ma et al., 2017).

**TABLE 2:** The properties of the standard protein complexes.

Datasets	Number	Protein coverage	Avg size
standard protein complexes 1	812	2,773	8.92
standard protein complexes 2	1,045	2,778	8.97

#### 2.1.3 GO Annotation Data and Gene Expression Data

In this study, we used the GO-slim data for describing the functional similarity of interactions, which is available on the link: https://downloads.yeastgenome.org. Meanwhile, the gene expression data were obtained from https://www.ncbi.nlm.nih.gov/sites/GDSbrowser. Additionally, subcellular localization data was obtained from https://compartments.jensenlab.org/Downloads.

**Algorithm 1:** The framework of ELF-DPC algorithm.

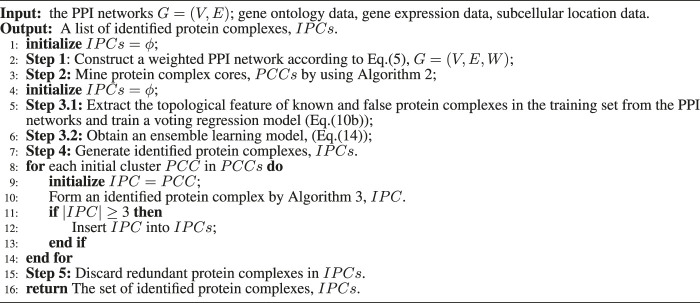

### 2.2 Terminologies

Here, we will give some terminologies that are used in this paper. A PPI network is generally described as a weighted graph *G* = (*V*, *E*, *W*), where *V* is a set of proteins, *E* is a set of interactions, and *W* is a *n* × *n*(*n* = |*V*|) matrix that represents the reliability of protein pairs in PPI networks. The direct interacting neighbor of node *v* is defined as *N*_*v*_ = {*u*|(*u*, *v*) ∈ *E*, *u* ∈ *V*}.

### 2.3 Methods

#### 2.3.1 The Framework of ELF-DPC Algorithm

**FIGURE 1:**
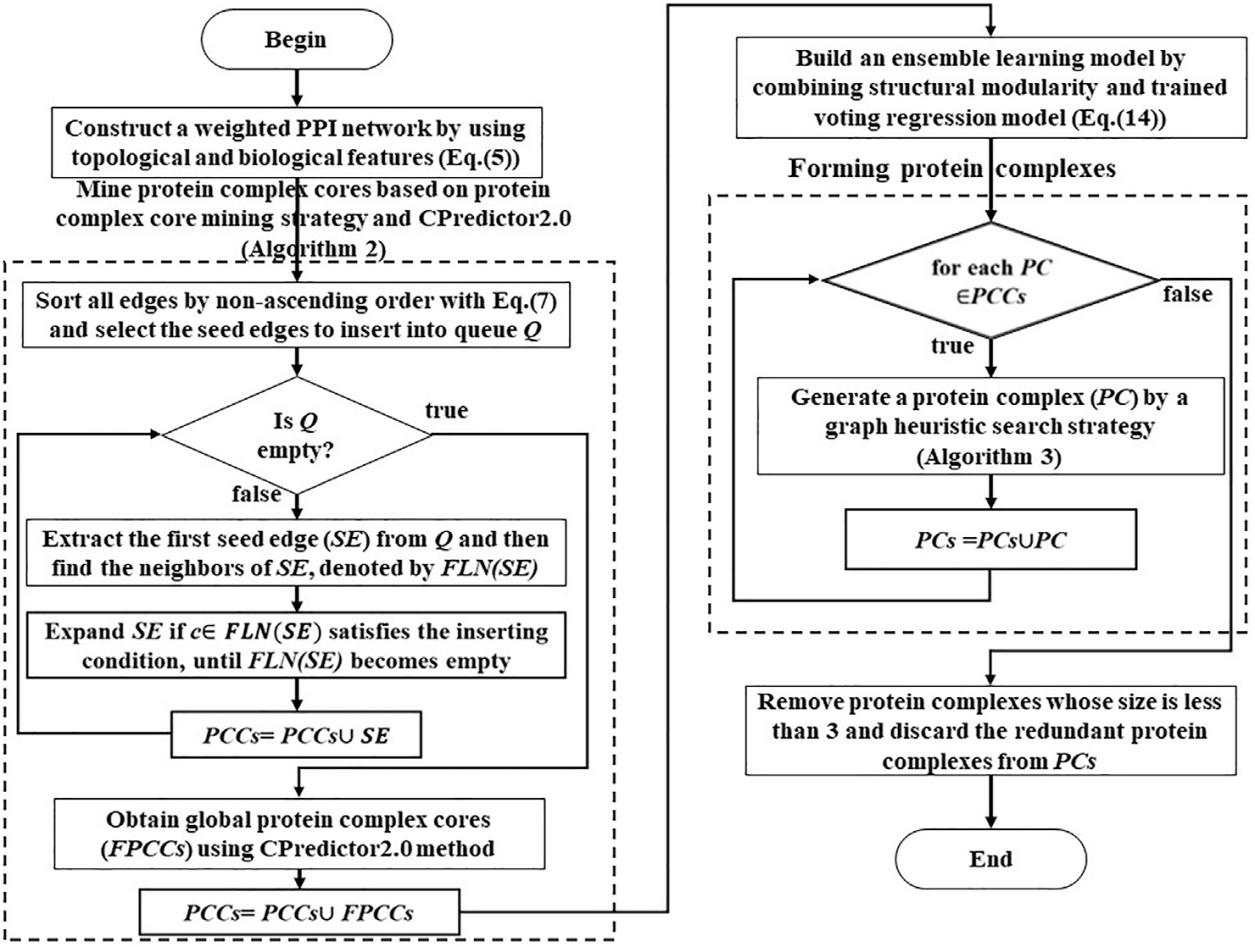
The ensemble framework of proposed protein complex detection.

This work is a novel ensemble learning framework to identify protein complexes from PPI networks. The block diagram of the detection process is shown in Figure 1.

The framework of this method is outlined in Algorithm 1. The input to the algorithm is the PPI network, which produces a set of protein complexes as output. Our algorithm consists of five main steps. The first step is to construct a weighted PPI network by combining topological structure, gene expression data, GO annotation data, and subcellular location data in Line 2 (Constructing a weighted PPI network section). The second step is to design a protein complex core mining strategy to identify protein complex cores in the PPI networks (Mining protein complex cores section) in Line 3. The third step is first to construct feature vectors to describe the properties of known and false protein complexes in the PPI networks and train a voting regression model (Training a voting regression model section) to model and represent the protein complex based on supervised learning in Line 5. Then second, we define a quality function called structural modularity to describe the structural modularity of protein complexes. Then we combine the trained voting regression model and structural modularity to obtain an ensemble learning model in Line 6. In the fourth step, based on the ensemble learning model, we propose a graph heuristic search strategy (Forming protein complexes section) to extend each protein complex core for forming protein complexes from the PPI networks in Lines 7–14. Finally, we remove these redundant identified protein complexes in Line 15.

#### 2.3.2 Constructing a Weighted PPI Network

Some studies have confirmed that the performance of protein complex detection could be markedly enhanced when the weight of edges is considered (Keretsu and Sarmah, 2016; Lei et al., 2018). Meanwhile, integrating multiple data sources into a PPI network can strengthen the reliability of the PPI networks (Lei et al., 2018; Wang et al., 2020), which inspires us with confidence to give the weight for interactions. Moreover, a protein complex consists of proteins and interactions among themselves, and the proteins in the same protein complex are coexpressed and have a similar function and localization. Thus, we integrate multiple pieces of information, including gene expression data, protein localization data, and gene ontology data, to weight the interactions within the PPI networks.

##### 2.3.2.1 Protein Coexpression Similarity

Generally, for a pair of interacting proteins, their coexpression level can reflect the strength of their interactions. Proteins with coexpressed relationships may also have similar functions (Eisen et al., 1998) and show stronger consistency of functions (Chen and Xu, 2004). Some studies have shown that coexpressed protein pairs tend to interact in the same protein complexes (Keretsu and Sarmah, 2016). Furthermore, the Person correlation coefficient (PCC) was used to estimate how strongly two interacting proteins are coexpressed (Lei et al., 2016; Shang et al., 2016). For a pair of proteins *X* and *Y*, their gene expression profiles are *X* = {*x*_1_, *x*_2_, … , *x*_*i*_, … , *x*_*m*_} and *Y* = {*y*_1_, *y*_2_, … , *y*_*i*_, … , *y*_*m*_}, respectively. The value of their PPC is defined as Eq. 1 (Wang et al., 2013).

$$PCC\left(X,Y\right)=\frac{\sum_{i=1}^{m}\left(x_{i}-\bar{X}\right)\times \left(y_{i}-\bar{Y}\right)}{\sqrt{\sum_{i=1}^{m}{\left(x_{i}-\bar{X}\right)}^{2}}\times \sqrt{\sum_{i=1}^{m}{\left(y_{i}-\bar{Y}\right)}^{2}}}$$ (1)

where $\bar{X}$ and $\bar{Y}$ are the average gene expression of proteins *X* and *Y* at *n* time points, respectively. The value of *PCC*(*X*, *Y*) ranges from -1 to 1. For convenience, we use (*PCC*(*X*, *Y*) + 1)/2 to replace *PCC*(*X*, *Y*), which sets the value of *PCC*(*X*, *Y*) in (0,1). The value of *PCC*(*X*, *Y*) is higher, and then the coexpression probability of nodes *X* and *Y* is larger. At the same time, they could consist of the same protein complex.

##### 2.3.2.2 Protein Functional Similarity

From a functional standpoint, we use GO-slim data to reflect the functional similarity of proteins. If a pair of proteins have more common GO-slim annotations, they are more likely to have the same biological function. Even the reliability of interactions between them will become stronger. Here, we let *FS*(*X*, *Y*) describe this relationship, which is defined as Eq. 2:

$$FS\left(X,Y\right)=\left\{\begin{array}{cc} \frac{\left|FS\left(X\right)\cap FS\left(Y\right)\right|}{\min\left\{\left|FS\left(X\right)|,|FS\left(Y\right)\right|\right\}}, & \min\left\{\left|FS\left(X\right)|,|FS\left(Y\right)\right|\right\}⩾1\\ 0,\; & otherwise \end{array}\right.$$ (2)

where |*FS*(*X*)| and |*FS*(*Y*)| represent the number of GO-slim annotations for proteins *X* and *Y*, respectively. |*FS*(*X*) ∩ *FS*(*Y*)| denotes the number of common GO-slim annotations for proteins *X* and *Y*.

##### 2.3.2.3 Protein Subcellular Location Similarity

Generally, if two interacting proteins have more exact subcellular locations, the interaction between proteins is more reliable. Here, we define the subcellular location similarity *SL*(*X*, *Y*), which is defined as Eq. 3:

$$SL\left(X,Y\right)=\frac{2\times |SL\left(X\right)\cap SL\left(Y\right)|}{\left|SL\left(X\right)|+|SL\left(Y\right)\right|}$$ (3)

where |*SL*(*X*)| and |*SL*(*Y*)| denote the number of subcellular localizations of proteins *X* and *Y*, respectively. |*SL*(*X*) ∩ *SL*(*Y*)| represents the number of common subcellular localizations between proteins *X* and *Y*.

##### 2.3.2.4 Protein Topological Structure Similarity

The network embedding method is a representation learning technique for representing the network’s nodes, which can automatically learn topological information from PPI networks. In this study, we use the network embedding method Node2Vec (Grover and Leskovec, 2016) to learn low-dimensional feature representations for the structural information of the proteins in a PPI network. For proteins *X* and *Y*, their representations are two vectors, namely, *X* and *Y*. Meanwhile, the obtained protein embedding vectors by node2vec can reflect the topological structure similarity among proteins, and we use cosine similarity to calculate the similarity of vector representation of proteins *X* and *Y*, which is defined as Eq. 4:

$$TSS\left(X,Y\right)=\frac{\sum_{i=1}^{n}x_{i}\times y_{i}}{\sqrt{\sum_{i=1}^{n}x_{i}^{2}}\times \sqrt{\sum_{i=1}^{n}y_{i}^{2}}}$$ (4)

where *F*(*X*) = (*x*_1_, *x*_2_, … , *x*_*i*_, … , *x*_*n*_) and *F*(*Y*) = (*y*_1_, *y*_2_, … , *y*_*i*_, … , *y*_*n*_) is the *n* dimension of the corresponding vector. *TSS*(*X*, *Y*) indicates the topological structure similarity of two connecting proteins, *X* and *Y*.

For each edge, its weighted value *W*(*X*, *Y*) is expressed by Eq. 5:

$$W\left(X,Y\right)=\frac{PCC\left(X,Y\right)+FS\left(X,Y\right)+SL\left(X,Y\right)+TSS\left(X,Y\right)}{4}$$ (5)

when the edges, whose weight is 0, are noise and should be removed from the PPI networks. Finally, we integrate topological structure similarity and biological information similarity, which can enhance the reliability of PPI networks. Therefore, a weighted PPI network is constructed.

#### 2.3.3 Mining Protein Complex Cores

According to the constructing a weighted PPI network section, the weight of interactions is weighted using multiple biological properties and its topological structure, so the higher weight the edge has, the more likely it is that two terminate proteins are inside the same protein complex (Wang et al., 2011; Li et al., 2012). Furthermore, the protein complex cores often correspond to dense subgraphs in PPI networks (Wu et al., 2009; Wang et al., 2019). The pseudocode of mining protein complex cores is presented in Algorithm 2.

First, for the edge (*v*, *u*), its weight is *w*(*v*, *u*), and its neighborhood graph is denoted as *NG*(*v*, *u*) = (V*, E*, W*), where V* = *N*_*v*_ ∪ *N*_*u*_ ∪ {*v*, *u*}. Furthermore, the average weighted degree of *NG*(*v*, *u*) is denoted as *AWD*(*NG*(*v*, *u*)) (Eq. 6):

$$AWD\left(NG\left(v,u\right)\right)=\frac{2\times \sum_{\left(s,t\right)\in E^{*}}w\left(s,t\right)}{\left|V^{*}\right|}.$$ (6)

Based on the analysis above, we propose a score function (Eq. 7) to score seed edges based on the weight of the edge *w*(*v*, *u*) and the average weighted degree of the neighborhood graph of the edge (Eq. 6) to select seed edges in Line 1. Then, we sort all edges in nonascending order based on the score function (see Eq. 7) in the PPI networks. Only edges whose score function is greater than the mean of the score function of all edges are queued into *Q*. Seed edges in *Q* will mine protein complex cores in Line 2.

As a result, the score function of edge (*v*, *u*) is defined as Eq. 7:

$$Score_{edge}\left(v,u\right)=w\left(v,u\right)\times AWD\left(NG\left(v,u\right)\right).$$ (7)

For an edge (*v*, *u*) ∈ *E*, its edge clustering coefficient (*ECC*(*v*, *u*)) is defined as the number of triangles to which (*u*, *v*) belongs, divided by the number of triangles that might potentially include (*u*, *v*), as shown in Eq. 8.

$$ECC\left(v,u\right)=\frac{Z\left(v,u\right)}{\min\left(\left|\deg\left(v\right)|,|\deg\left(u\right)\right|\right)}.$$ (8)

where *Z*(*v*, *u*) denotes the number of triangles built on edge (*v*, *u*), and *min(| deg*(*v*)|, | *deg*(*u*)|) is the minimum degree of the two terminate proteins.

Initially, select the protein with the highest weight edge as the first seed edge (*v*, *u*), and create a protein complex core in Line 6, where neighbors of the complex core are added to both the weight of edge *w*(*x*, *t*) ≥ *Avgedgesweight* (*Avgedgesweight* is defined as Eq. 9) and *ECC*(*x*, *t*) is greater than the average edge clustering coefficient *ECC* of all edges (*AvgweightECC*), according to the closeness between the seed edge (*v*, *u*) and its neighbors in Lines 9–17. These two constraints can ensure that the proteins in the protein complex core are correlated in biological relations and closely connected in topological structure. The protein complex core is retained if it contains more than or equals two proteins in Lines 18–20. Meanwhile, the seed edge (including two terminate proteins) would be marked and cannot be used as the seed edge of another cluster in Lines seven and eight. We select the next edge with the highest weight where its two terminal proteins are not included before seed edges, and it is used to form the next protein complex core until the seed queue *Q* is empty in Lines 6–22.

$$Avgedgesweight=\frac{\sum_{\left(v,u\right)\in E}w\left(v,u\right)}{\left|V\right|}.$$ (9)

CPredictor2.0 (Xu et al., 2017) is also employed to detect global protein complex cores. Here, CPredictor2.0 detects protein complexes using MCL and protein functional information. It first discovers clusters in each functional group using the Markov clustering algorithm and merges them with higher overlap. We use CPredictor2.0 to obtain global protein complex cores (*CPrclusters*) in Line 23. Next, we combine these local protein complex cores by a graph heuristic search method and global protein complex cores using the CPredictor2.0 method in Line 24.

Here, Algorithm 2 identifies the protein complex cores, which may have some redundant protein complex cores. For these redundant protein complex cores, we only keep one of them in the list of protein complex cores in Line 25.

Algorithm 2 Mining protein complex cores.

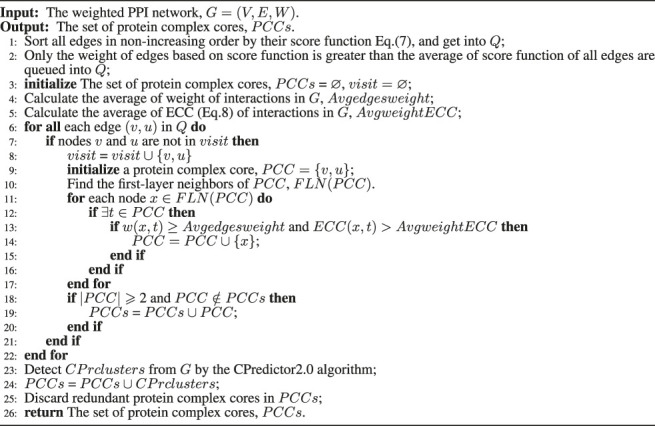

#### 2.3.4 Obtaining an Ensemble Learning Model

##### 2.3.4.1 Training a Voting Regression Model

**FIGURE 2:**
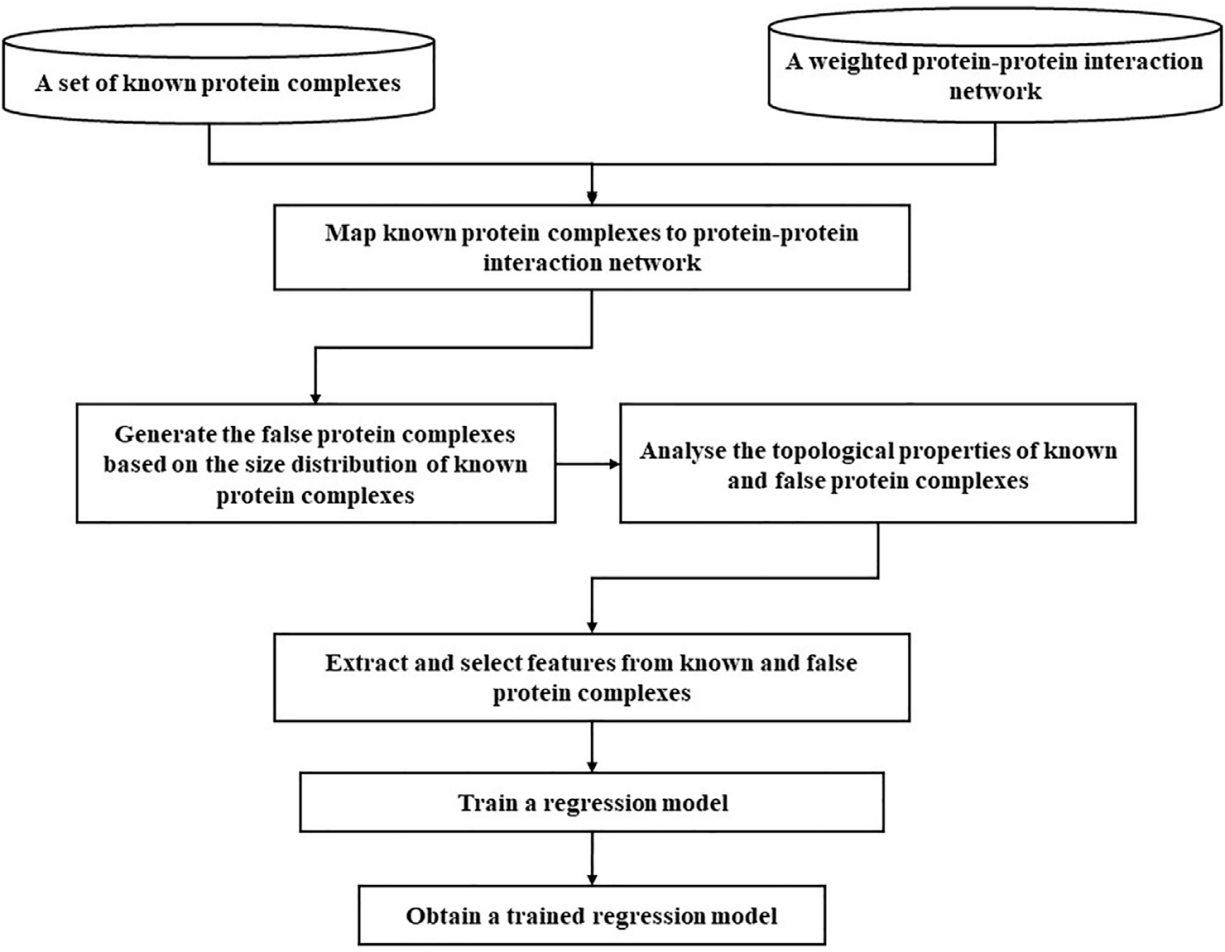
A procession of training a regression model.

To obtain the trained regression model, we will follow several steps. First, we collect the known protein complexes and weighted a weighted PPI network based on Eq. 5. Second, we map these known protein complexes to the weighted and unweighted PPI networks to obtain mapped protein complexes. Third, we generate false protein complexes in current weighted and unweighted PPI networks based on the same size distribution of mapped protein complexes. Then we analyze the topological properties of known and false protein complexes. Fourth, we extract and select topological features from these mapped protein complexes and false protein complexes. Fifth, we chose an appropriate regression model and train it. Finally, we obtained the trained regression model. The whole training routine is illustrated in Figure 2.

Next, we mainly introduce the differences and contributions between this study and previous research works. Obtaining known protein complexes from the database of standard protein complexes 1 and 2 (Wang et al., 2020) is very important, because they are used as factual protein complexes for training a model. Note that the protein complex has more than or equal to three proteins. Given machine learning, the quality of the training dataset is vital to model training. Previous methods generally construct false protein complexes by randomly selecting nodes in the graph. It has two disadvantages: it does not guarantee that the generated subgraphs are connected graphs and they cannot reflect the veracity of the topology of subgraphs in PPI networks. Therefore, we propose a false protein complex generating strategy. First, standard protein complexes are mapped to the PPI networks. Note that some standard protein complexes could not be mapped to the PPI networks, so the number of mapped protein complexes is generally less than the number of standard protein complexes. Second, we analyze the size distribution of the mapped protein complexes, and the size distribution of the generated false protein complexes follow the same power-law distribution. Third, according to the size distribution of the mapped protein complexes, we generate false protein complexes by randomly selecting the local neighborhood subgraphs in the PPI networks. Here, false protein complexes whose neighborhood affinity *NA*(*A*, *B*) (Eq. 15) with known protein complexes is less than 0.2. Finally, the ratio between the number of false protein complexes and the number of mapped protein complexes was 5 to 1. For selecting the parameter *ratio*, please see the parameter selection section.

In this paper, both known and false protein complexes in the PPI networks are modeled as weighted and unweighted undirected graphs. The weight is calculated based on Eq. 5. Extracting and selecting appropriate features are essential to distinguish between factual and false protein complexes. Previous supervised learning methods rely on finding cliques, triangles, rectangles, spokes, and star graphs to mine protein complexes in PPI networks. Of course, we can use topological features such as degree statistics, node size, and edge statistics. On the one hand, we use some existing topological features for protein complex identification.

On the other hand, we propose some topological features to describe the topological properties of protein complexes. We use 65 topological features to represent protein complexes in the PPI networks. Table 3 presents the list of topological features we used. Some topological features are extracted from the unweighted and weighted PPI networks. The implementation details about these topological features are well described in https://github.com/RongquanWang/ELF-DPC/Methods/Feature_selection.py. Additionally, if the reader discovers other relevant and valid topological features, please use them to represent protein complexes further.

**TABLE 3:** The topological features are used for representing protein complexes.

Num	Feature name	Num	Feature name
1	Graph entropy	2	Graph weight entropy
3	Node size	4	Edge size
5	Graph clustering coefficient	6	Maximum degree
7	Minimum degree	8	Mean degree
9	Median degree	10	Variance degree
11	standard deviation degree	12	Maximum weight degree
13	Minimum weight degree	14	Average weight degree
15	Median weight degree	16	standard weight degree
17	Graph density	18	Graph weight density
19	Edge mean weight	20	Edge median weight
21	Edge variance weight	22	Edge standard weight
23	Average shortest path length	24	Graph diameter
25	Maximum Clustering Coefficient	26	Minimum Clustering Coefficient
27	Mean Clustering Coefficient	28	Median Clustering Coefficient
29	Variance Clustering Coefficient	30	Graph conductance
31	Graph weight conductance	32	Modularity score
33	Weight modularity score	34	Average boundary edge weight
35	Average edge modularity	36	Average common neighbor
37	Standard common neighbor	38	Variance common neighbor
39	Minimum common neighbor	40	Median common neighbor
41	Maximum common neighbor	42	Mean topological features
43	Median topological feature	44	Variance topological feature
45	Maximum topological feature	46	Minimum topological feature
47	Standard topological feature	48	Mean Degree correlation
49	Minimum Degree correlation	50	Variance Degree correlation
51	Maximum Degree correlation	52	Median Degree correlation
53	Community model	54	Weight community model
55	Topological Change 1	56	Topological Change 2
57	Topological Change 3	58	Topological Change 4
59	Topological Change 5	60	Topological Change 6
61	Topological Change 7	62	Topological Change 8
63	First Eigenvalues 1	64	First Eigenvalues 2
65	First Eigenvalues 3		

Ensemble learning combines multiple individual learners with certain strategies to form a learning committee, so that the overall generalization performance is greatly improved. In general, the generalization capability of an ensemble learner model is much greater than the generalization capability of a single learner model. Meanwhile, we know that there is a barrel theory so we focus on two major standards: accuracy and diversity:

• Accuracy: The individual learner must not be too bad, but it must be accurate.
• Diversity: The output of individual learners should be different from each other.

Therefore, producing and combining “good but different” individual learners is the core of ensemble learning. The VotingRegressor model is one of the most efficient ensemble learning techniques to reduce the variance and improve detection accuracy. In this paper, we use a VotingRegressor model based on several base models for training. A VotingRegressor is an ensemble meta-estimator that fits several base estimators and averages the individual predictions to form a final prediction. Here, linear regression, BayesianRidge, DecisionTreeRegressor, and SVM. SVR (kernel = “linear”) are used as the base estimators to build the VotingRegressor model. We select the VotingRegressor model due to its reduced variance in individual base estimators and better generalization capabilities, and the Voting Regressor model has more robustness than a single estimator. In this study, the VotingRegressor model and base estimators use default parameters. These models are a freely available machine learning tool used on scikit-learn (Pedregosa et al., 2011), and they can be determined by the website https://scikit-learn.org/stable/supervised_learning.html*#*supervised-learning.

As a result, a trained VotingRegressor model could be used to estimate the probability of a subgraph being a natural protein complex from a supervised learning perspective to detect protein complexes with various topological structures. The score of the VotingRegressor is based on the higher probability that it is an actual protein complex. The VotingRegressor is defined as Eq. 10a and Eq. 10b:

$$\begin{array}{c} LR=LinearRegression\left(\right)\\ BSR=BayesianRidge\left(\right)\\ DTR=DecisionTreeRegressor\left(\right)\\ SVR=SVM.SVR\left(kernel{=}^{\prime }linear^{\prime }\right) \end{array}$$ (10a)

$$VR\left(C\right)=VotingRegressor\left(\left[\left(LR\right),\left(BSR\right),\left(DTR\right),\left(SVR\right)\right]\right)$$ (10b)

##### 2.3.4.2 The Structural Modularity of Protein Complexes

Based on the within-module and between module edges of subgraphs and the size of the subgraph, we present a new formal definition of protein complexes in PPI networks (Wu et al., 2009; Yu et al., 2011; Nepusz et al., 2012; Wang et al., 2019). Given the new module definition, an effective method of quantitative measurement is introduced to estimate the likelihood of a cluster *C* = (*V*_*C*_, *E*_*C*_, *W*_*C*_) being a protein complex in the PPI network. We introduce a structural modularity (SM) model to estimate the likelihood of a cluster *C* = (*V*_*C*_, *E*_*C*_, *W*_*C*_) being a protein complex, which can detect both dense and sparser protein complexes in PPI networks. First, structural modularity (SM) is combined by *Cohesion*(*C*) and *Coupling*(*C*), and *Cohesion*(*C*) is defined as Eq. 11 and *Coupling*(*C*) is defined as Eq. 12.

$$Cohesion\left(C\right)=\frac{2\times W_{in}\left(C\right)}{sqrt\left(\left|C\right|\right)\times \left(|C|-1\right)},$$ (11)

where $W_{in}=\sum_{\left(v,u\right)\in E_{C}}w\left(v,u\right)$ denotes the total weight of the internal edges contained entirely in cluster *C*, and |*C*| is the number of nodes in the cluster *C*. *Cohesion*(*C*) could estimate a protein complex with a community structure having dense connections among its nodes. Here, *Cohesion*(*C*) is based on the definition of density of a cluster *C* by density multiplied by the square root of the size of cluster *C* to quantify the likelihood that a cluster is a protein complex. The idea of *Cohesion*(*C*) is that a protein complex in the PPI network is usually relatively sparse, so *Cohesion*(*C*) is used to adopt density as the quality function, and it may be more appropriate.

$$Coupling\left(C\right)=\frac{W_{out}\left(C\right)}{\left|C\right|},$$ (12)

where *W*_*out*_(*C*) = *∑*_*v*∈*C*,*u*∉*C*_ *w*(*v*, *u*) represents the total weight of the boundary edges that connect the cluster *C* with the rest of the PPI network, and it can measure that the cluster *C* has sparse connections with its neighbor nodes.

Finally, Structural Modularity (SM) is calculated as Eq. 13:

$$SM\left(C\right)=\frac{Cohesion\left(C\right)}{Cohesion\left(C\right)+Coupling\left(C\right)}$$ (13)

In this work, a protein complex will be assigned a higher value of *SM*(*C*) when it has a high adapting density and is well separated from the rest of the network. *SM*(*C*) can identify protein complexes with cohesion and separation topological properties. This shows that proteins in a protein complex displayed intense and frequent connections within the protein complex and weak and rare connections to proteins outside of the protein complex.

##### 2.3.4.3 Building an Ensemble Learning Model

In this paper, we propose an ensemble learning model that combines the VotingRegressor model and structural modularity (SM) to quantify the likelihood of a cluster *C* = (*V*_*C*_, *E*_*C*_, *W*_*C*_) being a candidate protein complex to guide the identification of protein complex processes. An ensemble learning model can improve the robustness and stability of the clusterings by combining the output of several models, thus improving the overall accuracy. For a cluster *C*, its ensemble learning model is defined as Eq. 14:

$$Fitness\left(C\right)=VR\left(C\right)\times SM\left(C\right)$$ (14)

Based on the ensemble learning model, we will introduce a graph heuristic search strategy by using the ensemble learning model to form protein complexes.

#### 2.3.5 Forming Protein Complexes

Based on the fact that a protein complex core and attachment proteins form a protein complex, we obtain some protein complex cores. Next, we extract the attachment proteins of each protein complex core and select reliable attachments cooperating with its protein complex core to form a protein complex. We design a graph heuristic search strategy for each protein complex core to extend the protein complex core to form a whole protein complex. First, it starts with a protein complex core, which iteratively inserts neighboring proteins into the protein complex core and then removes proteins from the protein complex core to search for a locally optimal cluster. In this paper, each protein complex core is subjected to a graph heuristic search strategy and an ensemble learning model to form a protein complex. The basic idea of a graph heuristic search strategy for a protein complex core is iteratively extended and corrected to form a protein complex by maximizing the score of the ensemble learning model (please see Obtaining an ensemble learning model section).

The pseudocode of the graph heuristic search strategy is shown in Algorithm 3, which consists of the following steps:

i Input a protein complex core.
ii Adding outer boundary proteins process in Lines 3–12: First, for the current protein complex core, we construct its outer boundary proteins set. We first obtain all directly connected neighbor proteins of the current protein complex core, and then we rank these neighbor proteins according to the number of shared proteins between the neighbor of the neighbor protein and current protein complex core. We discard the neighboring proteins with fewer than two common proteins to select high-quality candidate neighboring proteins. Then we select only half of the neighboring protein set reserved according to the sorting results as the outer boundary proteins set in Line 3. Second, we calculate the ensemble learning model score for the current protein complex core when each outer boundary protein is temporarily added. The outer boundary protein that allows the ensemble learning model score to reach a maximum will be inserted into the protein complex core in Lines 5–11. This process is repeated until the ensemble learning model score of the protein complex core is not increased, or the size of the outer boundary nodes is zero in Lines 10 and 4.
iii First, for the current protein complex core, inner boundary proteins are the set of proteins that belong to the protein complex core and connect at least one other protein in the PPI networks in Line 16. Second, we calculate the score of the ensemble learning model after each inner boundary node is temporarily removed from the protein complex core. The inner boundary protein that increases the ensemble learning model score is determined, and it will be eliminated from the protein complex core in Lines 19–21. This process is continued until the ensemble learning model score of the protein complex core reaches a maximum or the size of the inner boundary protein set is zero, and the number of current protein complex cores is less than or equal to 2 in Lines 22–23 and 17.
iv We repeat ii) and iii) until the protein complex core is no longer changed or no increment in the *Fitness*(*SG*) of the protein complex core in Lines 27–30, the current protein complex core is considered to be formed as a locally optimal cluster in Line 2–31, and then output it as a detected protein complex in Line 32.

Finally, we select the next protein complex core. Then we repeat this process using a graph heuristic search strategy (Algorithm 3) to extend the next protein complex core to form a protein complex until no seed edges remain. In the last step of the algorithm, some redundant protein complexes and protein complexes containing fewer than three proteins are discarded.

Algorithm 3 A graph heuristic search strategy

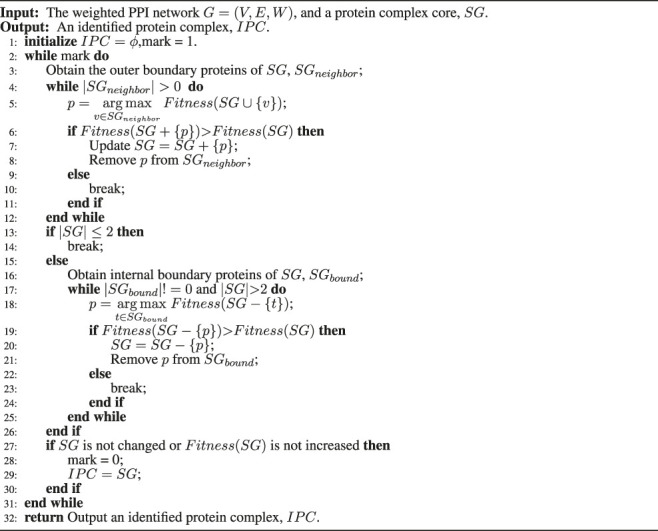

## 3 Experiments and Results

ELF-DPC was implemented in Python three and was successfully executed on a PC with an Intel i7-4790 CPU @3.60 GHz and 80 GB RAM.

### 3.1 Evaluation Metrics

In this study, to evaluate the proposed method, we need to compare the performance of our method against the compared methods by some statistical metrics. For this purpose, we used the neighborhood affinity, F-measure, CR, ACC, MMR, and Jaccard criteria to evaluate the protein complex detection algorithms. Let *S* denote the known protein complexes, and *D* denote the protein complexes identified by a detection method.

#### 3.1.1 Neighborhood Affinity

*S*_*i*_ is a standard protein complex in *S*, and *D*_*j*_ is a discovered protein complex *D*. Their neighborhood affinity score (*NA*(*S*_*i*_, *D*_*j*_)) (Brohee and Van Helden, 2006) can describe the similarity of two protein complexes *S*_*i*_ and *D*_*j*_, and it is defined as Eq.15:

$$NA\left(S_{i},D_{j}\right)=\frac{|S_{i}\cap D_{j}{|}^{2}}{\left|S_{i}|\times |D_{j}\right|}.$$ (15)

Generally, if *NA*(*S*_*i*_, *D*_*j*_) is larger than or equal to 0.2, protein complexes *S*_*i*_ and *D*_*j*_ are regarded as matching protein complexes (Li et al., 2010).

#### 3.1.2 F-Measure

Let *N*_*sm*_ be the number of standard protein complexes that match at least one detected protein complex, i.e., *N*_*sm*_ = |{*s*|*s* ∈ *S*, *∃d* ∈ *D*, *NA*(*s*, *d*) ≥ *ω*}| and *N*_*im*_ be the number of detected protein complexes that match at least one standard protein complex, i.e., *N*_*im*_ = |{*d*|*d* ∈ *D*, *∃s* ∈ *S*, *NA*(*d*, *s*) ≥ *ω*}|, where *ω* is a predefined threshold and is usually 0.20. Recall and precision are defined as $recall=\frac{N_{sm}}{\left|S\right|}$ and $precision=\frac{N_{im}}{\left|D\right|}$ , respectively. Finally, the F-measure is the compromise between precision and recall and is defined by Eq. 16:

$$F-measure=\frac{2\times precision\times recall}{precision+recall}.$$ (16)

#### 3.1.3 ACC

Let *T*_*ij*_ be the number of proteins that are included in both standard protein complex *S*_*i*_ and detected protein complex *D*_*j*_, and let *N*_*i*_ be the number of proteins that are included in standard protein complexes *S*. Meanwhile, Sn and PPV are calculated by $Sn=\frac{\sum_{i=1}^{\left|S\right|}\max_{j=1}^{\left|D\right|}\left\{T_{ij}\right\}}{\sum_{i=1}^{\left|S\right|}N_{i}}$ and $PPV=\frac{\sum_{j=1}^{\left|D\right|}\max_{i=1}^{\left|S\right|}\left\{T_{ij}\right\}}{\sum_{j=1}^{\left|D\right|}\sum_{i=1}^{\left|S\right|}T_{ij}}$ , respectively. As a result, the accuracy (ACC) is defined by Eq. 17:

$$\begin{array}{c} ACC=\sqrt{Sn\times PPV}. \end{array}$$ (17)

#### 3.1.4 MMR

We used the third metric, the maximum matching ratio (MMR) (Nepusz et al., 2012) based on the maximal one-to-one mapping between standard protein complexes and detected protein complexes. First, we need to construct a bipartite graph between *S* and *D*, and then each standard protein complex *S*_*i*_ ∈ *S* and detected protein complex *D*_*j*_ ∈ *D* are connected by the weight *W*(*S*_*i*_, *D*_*j*_) edge. Next, we select disjoint edges from the bipartite graph to maximize the sum of their weights; Finally, the MMR is the sum of the weights of all selected edges divided by |*S*|, which is denoted by Eq. 18:

$$MMR=\frac{\sum_{i=1}^{\left|S\right|}\underset{j}{\max}NA\left(S_{i},D_{j}\right)}{\left|S\right|}.$$ (18)

#### 3.1.5 Coverage Rate

The coverage rate (CR) was used to assess how many proteins in the standard protein complexes could be covered by the identified complexes. When the standard protein complexes *S* and the detected protein complexes *D* are given, the |*S*|×|*D*| matrix *T* is constructed, where each element max{*T*_*ij*_} is the most significant number of shared proteins between the *i*th standard protein complex, and the *j*th detected protein complex. The coverage rate is calculated by Eq. 19:

$$CR=\frac{\sum_{i=1}^{\left|S\right|}\max\left\{T_{ij}\right\}}{\sum_{i=1}^{\left|S\right|}N_{i}}.$$ (19)

where *N*_*i*_ is the number of proteins in the *i*th standard complex.

#### 3.1.6 Jaccard

Jaccard is the final method for measuring the clustering methods (Song and Singh, 2009). Here, a standard protein complex is *S*_*i*_ ∈ *S*, and a discovered protein complex is *D*_*j*_ ∈ *D*. Then, their Jaccard is $Jac\left(S_{i},D_{j}\right)=\frac{\left|S_{i}\cap D_{j}\right|}{\left|S_{i}\cup D_{j}\right|}$ . For the discovered protein complex *D*_*j*_, its Jaccard is $Jac\left(D_{j}\right)=max_{S_{i}\in S}Jac\left(D_{i},S_{i}\right)$ . For a standard protein complex *S*_*i*_, its Jaccard is $Jac\left(S_{i}\right)=max_{D_{j}\in D}Jac\left(S_{i},D_{j}\right)$ . Then, for detected protein complexes *D*, the average of the weighted Jaccard is $JaccardD=\frac{\sum_{D_{j}\in D}|D_{j}|Jac\left(D_{j}\right)}{\sum_{D_{j}\in D}|D_{j}|}$ . Similarly, for the standard protein complexes *S*, its JaccardS is defined by $JaccardS=\frac{\sum_{S_{i}\in S}|S_{i}|Jac\left(S_{i}\right)}{\sum_{S_{i}\in S}|S_{i}|}$ . Finally, the Jaccard is calculated by Eq. 20:

$$Jaccard=\frac{2\times \left(JaccardD\times JaccardS\right)}{JaccardD+JaccardS}.$$ (20)

#### 3.1.7 Functional Enrichment Analysis

In addition to these metrics to measure the performance of ELF-DPC, we investigated whether these identified protein complexes have biological significance by calculating the *p*-value. Generally, a detected protein complex possesses biological significance if its *p*-value is less than 0.01. In this paper, we used the fast tool LAGO (Boyle et al., 2004) to compute a *p*-value, and it is based on the hypergeometric distribution and Bonferroni correction. For more information about it, please refer to the literature (Boyle et al., 2004; Wang et al., 2019). The *p*-value is denoted as Eq. 21

p−value=1−∑i=0k−1FiN−FC−iNC, (21)

where *k* is the number of functional group proteins in the protein complex, and *N* is the number of proteins in the PPI networks. *F* is the size of the functional group in the PPI networks. We assume that a discovered protein complex contains *C* proteins.

### 3.2 Parameter Selection

**FIGURE 3:**
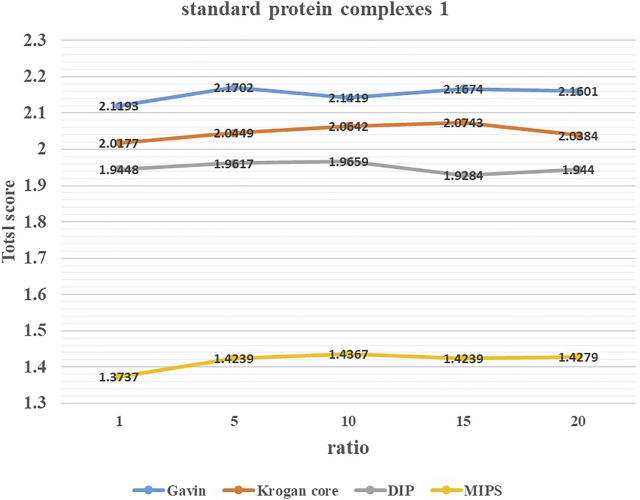
Value of parameters ratio for ELF-DPC based on standard protein complexes 1.

**FIGURE 4:**
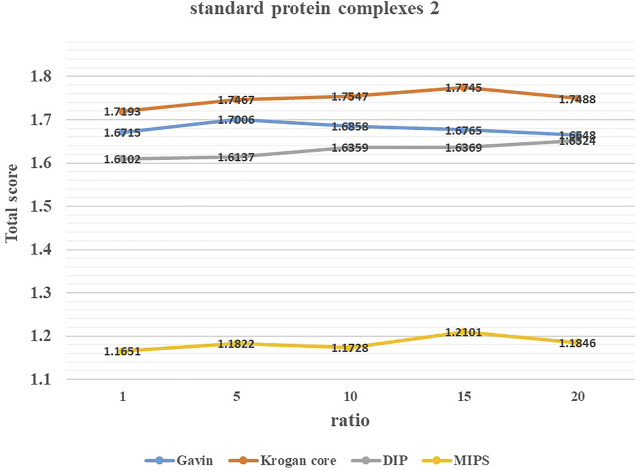
Value of parameters ratio for ELF-DPC based on standard protein complexes 2.

To study the effect of parameter *ratio* on the performance of ELF-DPC, we adjusted the value of *ratio* from 1 to 20 by increments of 5 through several experiments and set it to the appropriate values. Figures 3, 4 show the changing trend of the Total score with the value of *ratio* for the ELF-DPC algorithm with four PPI networks and two standard protein complex combinations. In standard protein complexes 1, *ratio* reaches its maximum value at *ratio* = 5. In standard protein complexes 2, *ratio* reaches its maximum value at *ratio* = 15. We can see that the Total score is not very sensitive to *ratio*, it tends to be stable when *ratio* falls in (5,15), and the fluctuations of the Total score are not significant. Therefore, the value of *ratio* is set as 5 by the default value in this study.

### 3.3 Comparison With State-of-the-art Algorithms

We obtained the software implementations for all the compared methods, and their parameters are shown in Table 4. Although better results could probably be obtained by fine-tuning these parameters, to maintain the fairness of different algorithms, the parameters of the compared algorithms and the ELF-DPC algorithm were set as the recommended values by the authors.

**TABLE 4:** Parameters of each method used in the study.

ID	Year	Algorithms	Parameter
1	2003	MCL	inflation = 2 (default setting)
2	2006	DPClus	*d*_*in*_ = 0.7, *cp*_*in*_ = 0.50 (author suggestions)
3	2009	CMC	min _*deg*_*ratio* = 1, min _*size* = 3, *overlap*_*thres* = 0.5, *merge*_*t*_ *hres* = 0.25(default setting)
4	2012	ClusterONE	Density = auto, Overlap threshold = 0.8(author suggestions)
5	2013	PEWCC	Overlap = 0.8,-r = 0.1, Re-join = 0.3(author suggestions)
6	2015	WPNCA	lambda = 0.3, size = 3 (author suggestions)
7	2016	CPredictor2.0	*func*_*lvl* = 6, Overlap threshold = 0.8, size = 3 (default setting)
8	2016	Zhang	*Complex*_*thresh* = 0.1 (author suggestions)
9	2017	ClusterEPs	NEPs of Complexes (minimum support threshold = 0.4, maximum support threshold = 0.05); NEPs of non-complexes (maximum support threshold = 0.05, minimum support threshold = 0.4); maximum overlap = 0.9, Maximum size of clusters = 100 (author suggestions)
10	2018	ClusterSS	numEpochs = 500, learnRate = 0.2, thresholdIn = 1.0, thresholdOut = 1.02, negativeTime = 20, minimum cluster size = 3 (author suggestions)
11	2019	ICJointLE	-L = 1,-r = 999,-d = 0.3,-c = 0.7,-f = 0.75,-p = 0.3,-m = 0.08, -u = 0.01,-e = 0.9, size = 3 (author suggestions)
12	2021	PC2P	minimum cluster size = 3
13	2022	ELF-DPC	*ratio* = 5, minimum cluster size = 3 (default setting)

In this section, we tested ELF-DPC on four original PPI networks, i.e., Gavin and Krogan core, DIP, and MIPS, and two known protein complexes were used for training and assessing the performance of ELF-DPC. We used six computational metrics, the F-measure, CR, ACC, MMR, Jaccard, and total score, to evaluate the performance. Here, we define the sum of the top five measures as the Total score. Note that the number of identified protein complexes (Num) was counted by each method. To illustrate the performance of ELF-DPC, we selected ten representative unsupervised methods, including DPClus (Altaf-Ul-Amin et al., 2006), CMC (Liu et al., 2009), ClusterONE (Nepusz et al., 2012), PEWCC (Zaki et al., 2013), WPNCA (Peng et al., 2014), CPredictor2.0 (Xu et al., 2017), Zhang (Zhang et al., 2016), ICJointLE (Zhang et al., 2019), PC2P (Omranian et al., 2021), and two state-of-the-art supervised methods, including ClusterEPs (Liu et al., 2016) and ClusterSS (Dong et al., 2018). Tables 5, 6 show the comparison results of all methods on four PPI networks in terms of six evaluation metrics, and the highest value of each metric of each PPI network is in bold.

**TABLE 5:** Experimental results by the different methods using standard protein complexes 1.

Name	Num	F-measure	CR	ACC	MMR	Jaccard	Total score
**Gavin**							
MCL	220	0.535 8	0.489 1	**0.365 7**	0.149 4	0.361 0	1.901 0
DPClus	285	0.597 2	0.438 2	0.346 6	0.173 6	0.402 5	1.958 1
CMC	294	0.584 4	0.450 1	0.348 7	0.222 9	0.417 9	2.023 9
ClusterONE	258	0.597 6	0.451 4	0.345 8	0.192 1	0.397 4	1.984 4
PEWCC	**664**	0.657 6	0.431 6	0.314 6	**0.353 8**	0.396 9	2.154 6
WPNCA	484	0.642 8	**0.494 9**	0.311 4	0.255 7	0.355 4	2.060 2
CPredictor2.0	266	0.628 6	0.375 0	0.306 2	0.214 4	0.412 4	1.936 5
Zhang	438	0.647 5	0.397 6	0.315 6	0.318 2	0.408 4	2.087 2
ClusterEPs	271	0.601 4	0.365 6	0.284 1	0.216 6	0.409 0	1.876 6
ClusterSS	482	0.560 0	0.394 1	0.321 8	0.253 5	0.368 5	1.897 9
ICJointLE	243	0.632 9	0.355 7	0.298 9	0.261 9	0.402 1	1.951 5
PC2P	219	0.576 9	0.443 9	0.355 1	0.182 5	0.392 2	1.950 5
ELF-DPC	286	**0.667 4**	0.479 2	0.339 1	0.251 6	**0.433 0**	**2.170 2**
**Krogan core**							
MCL	370	0.400 4	0.389 5	**0.319 2**	0.136 1	0.290 2	1.535 4
DPClus	497	0.413 8	0.367 2	0.307 1	0.174 5	0.323 5	1.586 1
CMC	264	0.481 9	0.365 6	0.297 8	0.158 4	0.368 8	1.672 4
ClusterONE	240	0.469 4	0.308 5	0.282 9	0.152 3	0.332 4	1.545 4
PEWCC	383	0.528 9	0.323 1	0.230 9	0.147 1	0.378 6	1.608 5
WPNCA	369	0.544 6	0.389 7	0.275 8	0.191 2	0.341 5	1.742 8
CPredictor2.0	236	0.589 5	0.303 7	0.272 5	0.195 4	0.368 8	1.729 8
Zhang	326	0.556 3	0.288 4	0.254 9	0.218 2	0.340 8	1.658 5
ClusterEPs	410	0.583 6	0.335 2	0.262 1	0.220 9	0.344 8	1.746 7
ClusterSS	**722**	0.437 7	0.375 8	0.307 2	0.240 2	0.335 7	1.696 6
ICJointLE	216	0.538 9	0.220 6	0.228 4	0.193 6	0.304 2	1.485 7
PC2P	249	0.435 6	0.345 8	0.297 0	0.133 7	0.319 0	1.531 0
ELF-DPC	304	**0.628 7**	**0.423 9**	0.298 4	**0.268 7**	**0.430 2**	**2.049 9**
**DIP**							
MCL	628	0.310 6	0.357 8	0.268 4	0.093 2	0.215 5	1.245 5
DPClus	909	0.308 5	0.379 2	0.272 0	0.123 7	0.264 5	1.348 0
CMC	1,192	0.361 1	0.355 2	0.248 8	0.197 3	0.296 0	1.458 4
ClusterONE	904	0.511 8	**0.506 2**	**0.327 0**	0.175 2	0.329 7	1.849 9
PEWCC	648	0.600 4	0.378 3	0.226 2	0.157 3	**0.351 4**	1.713 6
WPNCA	623	0.588 8	0.430 7	0.259 4	0.207 0	0.336 0	1.821 9
CPredictor2.0	293	0.500 8	0.230 2	0.228 7	0.111 0	0.282 5	1.353 3
Zhang	502	0.562 2	0.325 7	0.242 6	0.181 1	0.322 3	1.633 9
ClusterEPs	804	0.573 0	0.295 4	0.214 7	0.215 4	0.308 7	1.607 3
ClusterSS	**2,375**	0.323 0	0.333 5	0.257 7	**0.233 1**	0.257 3	1.404 7
ICJointLE	286	0.573 3	0.232 9	0.204 6	0.150 7	0.303 9	1.465 5
PC2P	441	0.341 9	0.340 1	0.254 2	0.085 4	0.232 4	1.254 0
ELF-DPC	564	**0.620 0**	0.492 2	0.276 8	0.227 3	0.345 4	**1.961 7**
**MIPS**							
MCL	594	0.068 1	0.168 6	0.157 7	0.021 4	0.106 4	0.522 1
DPClus	207	0.378 4	0.203 1	0.213 3	0.082 0	0.226 4	1.103 1
CMC	408	0.334 4	0.233 4	0.212 6	0.099 7	0.225 8	1.105 9
ClusterONE	690	0.292 5	0.271 9	**0.248 9**	0.098 9	0.204 4	1.116 7
PEWCC	382	0.280 2	0.190 0	0.138 9	0.056 6	0.167 9	0.833 5
WPNCA	527	0.330 1	0.260 3	0.182 4	0.101 7	0.179 8	1.054 3
CPredictor2.0	265	0.434 4	0.221 2	0.228 8	0.114 0	0.254 5	1.252 9
Zhang	406	0.370 2	0.205 1	0.202 5	0.107 7	0.217 6	1.103 1
ClusterEPs	645	0.461 0	0.242 6	0.194 3	0.158 0	0.254 3	1.310 2
ClusterSS	**1,266**	0.230 9	0.240 0	0.232 0	0.124 2	0.194 2	1.021 3
ICJointLE	121	0.364 9	0.134 3	0.172 3	0.084 5	0.206 6	0.962 6
PC2P	374	0.234 7	0.237 1	0.213 7	0.065 2	0.166 2	0.917 0
ELF-DPC	483	**0.481 1**	**0.291 4**	0.223 7	**0.167 8**	**0.259 9**	**1.423 9**

The bold values are the highest value of each metric of each PPI network.

**TABLE 6:** Experimental results by the different methods using standard protein complexes 2.

Name	Num	F-measure	CR	ACC	MMR	Jaccard	Total score
**Gavin**							
MCL	220	0.375 6	0.409 1	**0.358 7**	0.115 3	0.312 6	1.571 3
DPClus	285	0.385 4	0.348 3	0.329 3	0.140 5	0.314 7	1.518 2
CMC	294	0.380 3	0.357 5	0.330 1	0.145 9	0.325 7	1.539 5
ClusterONE	258	0.409 0	0.363 3	0.335 9	0.141 9	0.320 0	1.570 3
PEWCC	**664**	0.418 5	0.348 3	0.313 7	**0.215 2**	0.299 9	1.595 5
WPNCA	484	0.421 7	**0.411 6**	0.330 5	0.167 0	0.296 2	1.627 0
CPredictor2.0	266	**0.482 0**	0.307 6	0.281 6	0.156 4	0.330 9	1.558 4
Zhang	438	0.436 5	0.320 9	0.294 2	0.205 7	0.318 6	1.575 8
ClusterEPs	271	0.433 1	0.290 6	0.271 5	0.167 0	0.317 3	1.479 5
ClusterSS	487	0.372 9	0.327 9	0.317 0	0.171 6	0.292 4	1.481 9
ICJointLE	243	0.486 1	0.292 0	0.283 4	0.191 2	0.325 7	1.578 5
PC2P	219	0.402 5	0.361 0	0.341 3	0.129 5	0.320 4	1.554 7
ELF-DPC	265	0.454 6	0.383 8	0.325 9	**0.174 5**	**0.361 9**	**1.700 6**
**Krogan core**							
MCL	370	0.321 4	0.353 4	**0.308 8**	0.094 4	0.255 9	1.333 9
DPClus	**497**	0.357 7	0.333 5	0.289 9	0.120 0	0.289 3	1.390 4
CMC	264	0.399 9	0.319 2	0.273 2	0.110 1	0.314 9	1.417 3
ClusterONE	240	0.391 3	0.272 9	0.275 6	0.105 8	0.282 6	1.328 2
PEWCC	383	0.422 8	0.291 3	0.212 5	0.098 7	0.324 7	1.350 0
WPNCA	369	0.436 1	0.357 2	0.261 4	0.125 0	0.296 0	1.475 7
CPredictor2.0	236	0.493 2	0.278 7	0.242 1	0.125 8	0.321 6	1.461 4
Zhang	326	0.463 7	0.263 4	0.237 3	0.145 6	0.295 7	1.405 7
ClusterEPs	410	0.465 8	0.302 1	0.239 0	0.144 4	0.297 5	1.448 8
ClusterSS	342	0.430 4	0.320 1	0.270 5	0.131 8	0.314 0	1.466 9
ICJointLE	216	0.451 6	0.208 3	0.214 7	0.123 0	0.272 6	1.270 2
PC2P	249	0.363 6	0.314 1	0.288 4	0.095 1	0.281 8	1.342 9
ELF-DPC	281	**0.533 6**	**0.376 8**	0.282 7	0.175 0	**0.378 5**	**1.746 7**
**DIP**							
MCL	628	0.240 9	0.302 5	0.250 4	0.061 3	0.192 1	1.047 3
DPClus	909	0.278 4	0.342 4	0.249 3	0.089 8	0.244 5	1.204 4
CMC	1,192	0.313 0	0.321 3	0.219 3	0.132 9	0.266 4	1.253 0
ClusterONE	904	0.423 2	**0.435 8**	**0.293 7**	0.118 4	0.287 4	1.558 5
PEWCC	648	0.481 2	0.333 6	0.218 2	0.095 0	0.298 6	1.426 6
WPNCA	623	0.460 3	0.370 9	0.247 2	0.122 6	0.286 6	1.487 6
CPredictor2.0	293	0.465 3	0.226 5	0.207 7	0.073 6	0.263 5	1.236 7
Zhang	502	0.492 9	0.292 8	0.221 5	0.122 3	0.281 8	1.411 3
ClusterEPs	804	0.461 1	0.264 6	0.192 9	0.132 3	0.265 2	1.316 2
ClusterSS	**2,179**	0.367 6	0.316 8	0.236 0	0.158 8	0.234 0	1.313 2
ICJointLE	286	0.473 4	0.216 8	0.202 7	0.096 1	0.266 8	1.255 8
PC2P	441	0.266 2	0.296 7	0.233 7	0.058 8	0.208 3	1.063 6
ELF-DPC	545	**0.512 6**	0.399 8	0.260 7	**0.138 6**	**0.302 0**	**1.613 7**
**MIPS**							
MCL	594	0.055 1	0.164 0	0.147 5	0.012 5	0.103 1	0.482 2
DPClus	207	0.330 7	0.193 4	0.194 8	0.054 7	0.204 9	0.978 5
CMC	408	0.298 1	0.212 5	0.187 3	0.064 2	0.199 9	0.962 0
ClusterONE	690	0.247 3	0.238 4	**0.214 8**	0.063 0	0.180 1	0.943 5
PEWCC	382	0.230 9	0.170 0	0.116 6	0.029 6	0.130 1	0.677 3
WPNCA	527	0.264 0	0.238 3	0.154 9	0.062 1	0.152 2	0.871 6
CPredictor2.0	265	0.384 3	0.208 6	0.196 6	0.067 2	0.226 4	1.083 1
Zhang	406	0.341 3	0.194 4	0.185 7	0.071 0	0.200 2	0.992 5
ClusterEPs	645	0.358 2	0.211 5	0.172 0	0.088 4	0.212 0	1.042 1
ClusterSS	**1,581**	0.253 9	0.256 6	0.207 4	0.089 4	0.186 7	0.994 0
ICJointLE	121	0.295 9	0.122 4	0.159 3	0.053 8	0.178 7	0.810 1
PC2P	374	0.207 8	0.213 6	0.194 1	0.043 2	0.152 4	0.811 2
ELF-DPC	469	**0.402 6**	**0.259 9**	0.193 7	**0.101 1**	**0.224 9**	**1.182 2**

The bold values are the highest value of each metric of each PPI network.

As shown in Table 5, when standard protein complexes 2 was used as the training set and standard protein complexes 1 was used as the test set, the ELF-DPC achieved the highest F-measure, Jaccard, and Total score based on most of the four PPI networks. For the Gavin dataset shown in Table 5, the ELF-DPC algorithm ranks third in terms of CR, sixth in terms of ACC, and sixth in terms of MMR. The Krogan core dataset shown in Table 5 shows that the ELF-DPC achieves first place on CR and obtains four places on the ACC statistics. However, ELF-DPC achieves first place on MMR, it is 0.2687. For the DIP dataset shown in Table 5, the ELF-DPC method takes second in terms of CR and ACC metrics, the ELF-DPC algorithm has the second-highest top level in terms of MMR, and the ELF-DPC method takes second in terms of Jaccard, which is slightly lower than the best at 0.3454. For the MIPS dataset shown in Table 5, it can be seen that the ELF-DPC method takes first in terms of CR, at 0.2914. The ELF-DPC algorithm has the fourth-highest top level in terms of ACC, and the ELF-DPC algorithm is the first place in terms of MMR.

We used standard protein complexes 1 as the positive training set and standard protein complexes 2 as the test set. The results are presented in Table 6. One can quickly find that ELF-DPC has the best F-measure, MMR, Jaccard, and Total score on most tested datasets. Although ELF-DPC did not obtain the highest score in terms of CR, and ACC, the experimental comparison results are similar, taking standard protein complexes 1 in Table 5 as the test set. According to the experimental results in Tables 1 and 2, in some cases, some algorithms that identify more protein complexes achieve the highest MMR, such as PEWCC and ClusterSS, which means that detection algorithms that detect more protein complexes are suitable for MMR. Meanwhile, the number of protein complexes identified by the ELF-DPC algorithm is relatively small. However, it also achieves the highest values on some datasets, indicating that identifying protein complexes by the ELF-DPC algorithm can obtain a better maximal one-to-one mapping to standard protein complexes. On the whole, comparative experimental results show that ELF-DPC can achieve a higher Total score than all the compared methods on all datasets, which means that ELF-DPC performs better than these competitive methods on most computational evaluation metrics in the tested datasets.

### 3.4 Comparison With Functional Enrichment Analysis

We further substantiated the biological significance of the detected protein complexes by different methods by comparing the *p*-value of the identified proteins in GO (Gene Ontology) databases, which cover three domains: biological process, molecular function, and cellular component. Since the *p*-values of identified protein complexes are closely related to their size (Wang et al., 2019), we need to perform a comprehensive analysis of these statistics. Therefore, the number of significantly identified protein complexes and the percentage of them in different values of the *p*-value from 1E-2 to 1E-20 were used to estimate their functional enrichment. We analyzed the protein complexes discovered by ELF-DPC and compared algorithms using the *p*-value test. Generally, a protein complex with a lower *p*-value is significant. The functional enrichment analysis results for these methods are shown in Tables 7 and 8, where *Num* is the total number of identified protein complexes, and *AS* is the mean of the sizes of identified protein complexes.

**TABLE 7:** Results of function enrichment test with different thresholds of *p*-value on Gavin and Krogan core.

Algorithms	Num	As	< E-20	< E-15	< E-10	< E-5	Significant
**Gavin**							
MCL	220	7.56	39(17.73%)	48(21.82%)	83(37.73%)	183(83.18%)	194(88.18%)
DPClus	285	6.09	30(10.53%)	49(17.2%)	88(30.88%)	182(63.86%)	208(72.98%)
CMC	294	5.83	43(14.63%)	57(19.39%)	82(27.89%)	171(58.16%)	206(70.06%)
ClusterONE	258	7.24	39(15.12%)	53(20.55%)	101(39.15%)	187(72.48%)	205(79.46%)
PEWCC	**664**	8.14	61(9.19%)	117(17.62%)	238(35.84%)	480(72.29%)	546(82.23%)
CPredictor2.0	266	6.04	29(10.9%)	51(19.17%)	122(45.86%)	231(86.84%)	244(91.73%)
WPNCA	484	16.62	**125(25.83%)**	**180(37.19%)**	**281(58.06%)**	423(87.4%)	449(92.77%)
Zhang	438	6.30	44(10.05%)	83(18.95%)	164(37.44%)	318(72.6%)	354(80.82%)
ClusterEPs	271	6.25	53(19.56%)	86(31.74%)	143(52.77%)	**240(88.56%)**	**256(94.46%)**
ClusterSS	482	5.62	63(13.07%)	95(19.71%)	167(34.65%)	336(69.71%)	368(76.35%)
	487	5.36	50(10.27%)	83(17.05%)	147(30.19%)	324(66.53%)	368(75.56%)
ICJointLE	243	5.73	25(10.29%)	27(11.11%)	83(34.16%)	196(80.66%)	207(85.19%)
PC2P	219	6.91	17(7.76%)	11(5.02%)	40(18.26%)	106(48.4%)	119(54.34%)
ELF-DPC	286	8.81	59(20.63%)	104(36.36%)	154(53.84%)	244(85.31%)	262(91.6%)
	265	8.66	65(24.53%)	89(33.59%)	140(52.84%)	231(87.18%)	244(92.09%)
**Krogan core**							
MCL	370	5.91	82(22.16%)	119(32.16%)	173(46.75%)	275(74.32%)	293(79.18%)
DPClus	497	4.23	20(4.02%)	43(8.65%)	75(15.09%)	253(50.9%)	303(60.96%)
CMC	264	5.05	20(7.58%)	29(10.99%)	44(16.67%)	60(22.73%)	63(23.87%)
ClusterONE	240	5.27	44(18.33%)	75(31.25%)	121(50.42%)	202(84.17%)	216(90.0%)
PEWCC	383	10.16	**152(39.69%)**	**205(53.53%)**	**277(72.33%)**	**358(93.48%)**	**377(98.44%)**
CPredictor2.0	236	5.19	24(10.17%)	46(19.49%)	93(39.41%)	213(90.26%)	219(92.8%)
WPNCA	369	12.59	43(11.65%)	81(21.95%)	172(46.61%)	321(86.99%)	339(91.87%)
Zhang	326	5.41	37(11.35%)	65(19.94%)	118(36.2%)	259(79.45%)	279(85.58%)
ClusterEPs	410	6.18	59(14.39%)	95(23.17%)	168(40.97%)	341(83.17%)	365(89.02%)
ClusterSS	**722**	4.86	47(6.51%)	95(13.16%)	160(22.16%)	371(51.38%)	454(62.88%)
	342	7.01	48(14.04%)	88(25.74%)	155(45.33%)	280(81.88%)	304(88.9%)
ICJointLE	216	4.41	16(7.41%)	21(9.72%)	68(31.48%)	184(85.18%)	192(88.88%)
PC2P	249	5.81	16(6.43%)	23(9.24%)	46(18.48%)	136(54.62%)	159(63.86%)
ELF-DPC	304	9.55	80(26.32%)	115(37.83%)	163(53.62%)	277(91.12%)	292(96.05%)
	281	9.13	81(28.83%)	111(39.51%)	155(55.17%)	262(93.25%)	269(95.74%)

The bold values are the highest value of each metric of each PPI network.

**TABLE 8:** Results of function enrichment test with different thresholds of *p*-value on DIP and MIPS.

Algorithms	Num	As	< E-20	< E-15	< E-10	< E-5	Significant
**DIP**							
MCL	628	6.31	74(11.78%)	125(19.9%)	209(33.28%)	414(65.92%)	471(75.0%)
DPClus	909	4.28	45(4.95%)	64(7.04%)	112(12.32%)	364(40.04%)	470(51.7%)
CMC	1,192	3.81	90(7.55%)	150(12.58%)	304(25.5%)	692(58.05%)	829(69.54%)
ClusterONE	904	6.40	54(5.97%)	110(12.16%)	259(28.64%)	606(67.02%)	705(77.97%)
PEWCC	648	10.10	156(24.07%)	**249(38.42%)**	**379(58.48%)**	584(90.12%)	605(93.36%)
CPredictor2.0	293	4.54	18(6.14%)	49(16.72%)	124(42.32%)	274(93.51%)	**285(97.26%)**
WPNCA	623	12.41	81(13.0%)	137(21.99%)	228(36.6%)	431(69.18%)	481(77.21%)
Zhang	502	5.18	44(8.76%)	99(19.72%)	200(39.84%)	424(84.46%)	448(89.24%)
ClusterEPs	804	4.26	91(11.32%)	145(18.04%)	268(33.34%)	625(77.74%)	683(84.95%)
ClusterSS	**2,375**	3.57	156(6.57%)	253(10.65%)	437(18.4%)	1,047(44.08%)	1,289(54.27%)
	2,179	5.74	110(5.05%)	230(10.56%)	501(23.0%)	1,332(61.14%)	1,574(72.25%)
ICJointLE	286	3.84	29(10.14%)	27(9.44%)	103(36.01%)	248(86.71%)	253(88.46%)
PC2P	441	6.25	25(5.67%)	14(3.17%)	45(10.2%)	185(41.95%)	230(52.15%)
ELF-DPC	564	14.43	140(24.82%)	186(32.98%)	289(51.24%)	**512(90.78%)**	542(96.1%)
	545	12.77	**142(26.06%)**	203(37.25%)	307(56.33%)	493(90.46%)	517(94.86%)
**MIPS**							
MCL	594	6.16	17(2.86%)	29(4.88%)	80(13.47%)	165(27.78%)	230(38.72%)
DPClus	207	4.94	17(8.21%)	27(13.04%)	85(41.06%)	169(81.64%)	184(88.89%)
CMC	408	4.87	30(7.35%)	49(12.01%)	101(24.76%)	234(57.36%)	278(68.14%)
ClusterONE	690	6.03	22(3.19%)	47(6.81%)	137(19.85%)	327(47.39%)	483(70.0%)
PEWCC	382	24.70	67(17.54%)	94(24.61%)	172(45.03%)	308(80.63%)	325(85.08%)
CPredictor2.0	265	4.60	19(7.17%)	40(15.09%)	118(44.52%)	**249(93.95%)**	258(97.35%)
WPNCA	527	18.27	60(11.39%)	103(19.55%)	234(44.41%)	436(82.74%)	471(89.38%)
Zhang	406	5.14	16(3.94%)	37(9.11%)	111(27.34%)	319(78.57%)	355(87.44%)
ClusterEPs	645	4.78	22(3.41%)	45(6.98%)	150(23.26%)	443(68.69%)	500(77.53%)
ClusterSS	1,266	4.22	33(2.61%)	70(5.53%)	176(13.9%)	607(47.94%)	752(59.39%)
	**1,581**	5.81	25(1.58%)	67(4.24%)	237(14.99%)	845(53.45%)	1,069(67.62%)
ICJointLE	121	3.70	14(11.57%)	16(13.22%)	42(34.71%)	102(84.3%)	103(85.13%)
PC2P	374	6.29	7(1.87%)	4(1.07%)	41(10.96%)	171(45.72%)	202(54.01%)
ELF-DPC	483	9.33	**109(22.57%)**	**166(34.37%)**	246(50.93%)	441(91.3%)	463(95.85%)
	469	8.86	105(22.39%)	155(33.05%)	**253(53.95%)**	437(93.18%)	**458(97.66%)**

The bold values are the highest value of each metric of each PPI network.

As Table 7 shows, for the PPI Gavin dataset, ClusterEPs obtains a higher proportion of significantly identified protein complexes, which reaches 94.46*%*, higher than our ELF-DPC. However, ELF-DPC achieves a high proportion of significantly identified protein complexes with a *p*-value ≥ E-15. For the Krogan core PPI datasets, PEWCC attains a higher proportion of significantly identified protein complexes than our ELF-DPC. The reason is that ClusterEPs identifies the mean size of the identified protein complexes (*AS*) as 10.16. The *AS* of our ELF-DPC is 9.55 and 9.13, respectively. Generally, the *p*-value of an identified protein complex is closely associated with the size of the identified protein complex. Then the *p*-value decreases gradually when the size of the detected protein complexes increases (Wu et al., 2009; Peng et al., 2014). As Table 8 shows, for the PPI dataset DIP, CPredictor2.0 obtains a higher proportion of significantly identified protein complexes than our ELF-DPC. At the same time, ELF-DPC achieves a high proportion of significantly identified protein complexes with *p*-value ≥ E-20. For dataset MIPS, ELF-DPC performs better than other competing methods regarding the proportion of significantly identified complexes.

Therefore, we can conclude that ELF-DPC could detect more protein complexes with biological significance. Although some detected protein complexes currently do not match known protein complexes, they are more likely to be verified as actual protein complexes by laboratory techniques. Based on the above results, the protein complexes identified by ELF-DPC have significant biological meaning.

### 3.5 Case Study

**FIGURE 5:**
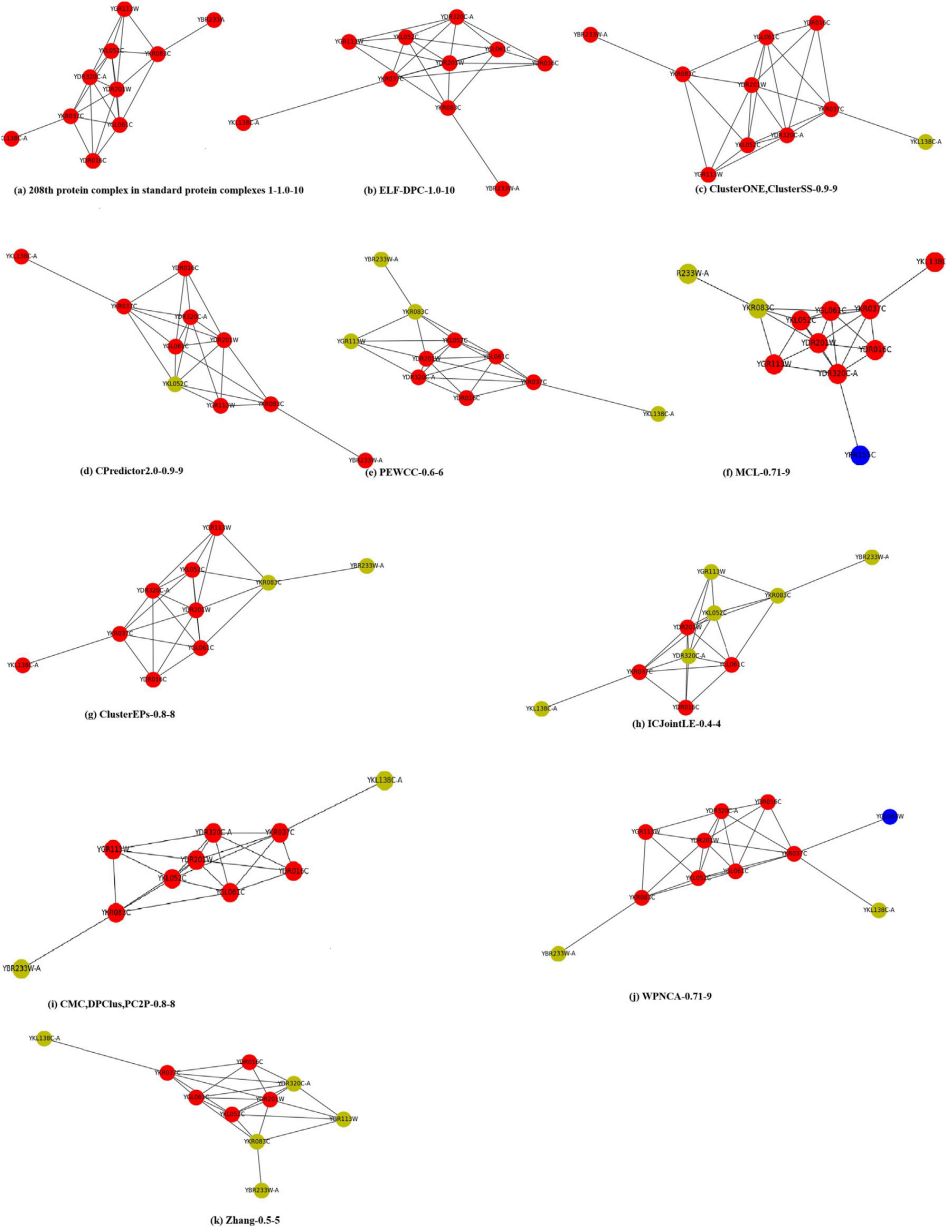
An example protein complex identified by different methods on the Krogan core PPI network. For example, (b) ELF-DPC-1.0–10, which means that the neighborhood affinity (Eq. 15) of ELF-DPC is 1.0, and it contains 10 proteins. Here, the red nodes are proteins that are correctly identified by this method, the yellow nodes are proteins that are missed by this method, and the blue nodes are the proteins that are incorrectly identified by this method.

To clearly show the clustering results, we visualized the 208th standard protein complex of standard protein complexes 1 in Figure 5. We define a format to allow readers to obtain information. For example, (b) ELF-DPC-1.0–10, which means that the neighborhood affinity (Eq. 15) of ELF-DPC is 1.0, and it contains 10 proteins. Here, the red nodes are proteins that are correctly identified by this method, the yellow nodes are proteins that are missed by this method, and the blue nodes are the proteins that are incorrectly identified by this method. Figure 5 (a) shows that there were 10 proteins in the 208th standard protein complex. The clustering results of the other thirteen methods (b) ELF-DPC, (c) ClusterONE and ClusterSS, (d) CPredictor2.0, (e) PEWCC, (f) MCL, (g) ClusterEPs, (h) ICJointLE, (i) CMC, DPClus, PC2P, (j) WPNCA, and (k) Zhang are all from the Krogan core dataset. (c) ClusterONE and ClusterSS, (d) CPredictor2.0, (e) PEWCC, (g) ClusterEPs, (h) ICJointLE, (i) CMC, DPClus, PC2P, and (k) Zhang only successfully identified part of the 208th standard protein complex, and they also did not identify some proteins. Meanwhile, (j) WPNCA and (f) MCL missed some proteins and incorrectly identified some proteins. However, our ELF-DPC method accurately identified 10 proteins and achieved the best performance in identifying the 208th standard protein complex.

Moreover, Table 9 provides 16 protein complexes with vital biological significance identified by the ELF-DPC algorithm in four PPI networks, which provide helpful biological knowledge to related researchers.

**TABLE 9:** The identified protein complexes with small *p*-values.

Num	*p*-value	GOID	Gene ontology term
**Gavin**			
1	9.72 641e-59	GO:0000 502	proteasome complex
2	4.53 112e-61	GO:0005 762	mitochondrial large ribosomal subunit
3	9.18 655e-68	GO:0030 686	90S preribosome
4	2.61 255e-65	GO:0030 532	small nuclear ribonucleoprotein complex
**Krogan core**			
1	2.50 943e-71	GO:0000 375	RNA splicing, *via* transesterification reactions
2	1.21 735e-66	GO:0005 681	spliceosomal complex
3	7.46 423e-67	GO:0000 377	RNA splicing, *via* transesterification reactions with bulged adenosine as nucleophile
4	5.5 331e-62	GO:0003 899	DNA-directed 5′-3′ RNA polymerase activity
**DIP**			
1	2.14 679e-64	GO:0042 254	ribosome biogenesis
2	5.5 228e-53	GO:0042 274	ribosomal small subunit biogenesis
3	5.18 295e-62	GO:0016 592	mediator complex
4	6.85 479e-66	GO:0097 525	spliceosomal snRNP complex
**MIPS**			
1	1.22 375e-47	GO:0050 657	nucleic acid transport
2	1.27 336e-44	GO:0030 687	preribosome, large subunit precursor
3	1.58 322e-42	GO:0022 624	proteasome accessory complex
4	9.71 714e-32	GO:0000 124	SAGA complex

## 4 Conclusion

Although many protein complex detection methods have been presented in the recent decades, the detection method with excellent performance is still a bottleneck in bioinformatics. This study presented an ensemble learning framework to identify protein complexes according to the core-attachment structure of protein complexes. First, a weighted PPI network was constructed by integrating the gene expression data, gene ontology data, and subcellular location data, as well as topological structure. Next, we used the protein complex core mining strategy to find protein complex cores. After that, we provided a new model training method to construct a training dataset and then extracted various topological features for training a VotingRegressor model to describe protein complexes based on supervised learning. Furthermore, we defined structural modularity for modeling the internal organization of protein complexes. As a result, an ensemble learning model is presented to guide the search for protein complexes. Finally, we designed a graph heuristic search strategy for extending protein complex cores to form protein complexes in the PPI networks. The experimental results show that ELF-DPC performs better than other competing methods. Moreover, our ELF-DPC can mine protein complexes with high biological significance. Because our ELF-DPC can not detect small protein complexes (size ≤2), we will consider integrating other data sources (Tan et al., 2018) to identify small protein complexes. In the future, we can infer drug-disease associations by constructing a heterogeneous network consisting of drugs, detected protein complexes, and diseases to unveil disease mechanisms, and discover available drugs (Yu et al., 2015). In addition, we also consider using graph attention networks and deep learning methods to identify protein complexes.

## Data Availability

The original contributions presented in the study are included in the article/Supplementary Material, further inquiries can be directed to the corresponding author.
